# Analyzing the cellular plasma membrane by fast and efficient correlative STED and platinum replica EM

**DOI:** 10.3389/fcell.2023.1305680

**Published:** 2023-11-30

**Authors:** Dmytro Puchkov, Paul Markus Müller, Martin Lehmann, Claudia Matthaeus

**Affiliations:** ^1^ Cellular Imaging Facility, Leibniz-Forschungsinstitut für Molekulare Pharmakologie, Berlin, Germany; ^2^ Institute for Chemistry and Biochemistry, Freie Universität Berlin, Berlin, Germany; ^3^ Cellular Physiology of Nutrition, Institute for Nutritional Science, University of Potsdam, Potsdam, Germany

**Keywords:** plasma membrane, endocytosis, CLEM, STED, TEM, SEM, electron microscopy

## Abstract

The plasma membrane of mammalian cells links transmembrane receptors, various structural components, and membrane-binding proteins to subcellular processes, allowing inter- and intracellular communication. Therefore, membrane-binding proteins, together with structural components such as actin filaments, modulate the cell membrane in their flexibility, stiffness, and curvature. Investigating membrane components and curvature in cells remains challenging due to the diffraction limit in light microscopy. Preparation of 5–15-nm-thin plasma membrane sheets and subsequent inspection by metal replica transmission electron microscopy (TEM) reveal detailed information about the cellular membrane topology, including the structure and curvature. However, electron microscopy cannot identify proteins associated with specific plasma membrane domains. Here, we describe a novel adaptation of correlative super-resolution light microscopy and platinum replica TEM (CLEM-PREM), allowing the analysis of plasma membrane sheets with respect to their structural details, curvature, and associated protein composition. We suggest a number of shortcuts and troubleshooting solutions to contemporary PREM protocols. Thus, implementation of super-resolution stimulated emission depletion (STED) microscopy offers significant reduction in sample preparation time and reduced technical challenges for imaging and analysis. Additionally, highly technical challenges associated with replica preparation and transfer on a TEM grid can be overcome by scanning electron microscopy (SEM) imaging. The combination of STED microscopy and platinum replica SEM or TEM provides the highest spatial resolution of plasma membrane proteins and their underlying membrane and is, therefore, a suitable method to study cellular events like endocytosis, membrane trafficking, or membrane tension adaptations.

## 1 Introduction

The plasma membrane of mammalian cells consists of a 5-nm-sized phospholipid bilayer that separates the intracellular environment from the outside of the cell (Sezgin et al., 2017). Therefore, the plasma membrane must ensure communication with neighboring cells and enhance sufficient exchange of nutrients and ions, as well as the uptake of signaling molecules or plasma membrane receptors (Cooper GM, 2000). Depending on the biological species, cell type, organelle, and subcellular localization, biological membranes consist of highly specific lipid species, allowing the recruitment of individual membrane-binding proteins (Harayama and Riezman, 2018). The localization of membrane proteins is highly specific and determines (sub-) cellular function and influences the overall physiology of the cell (Cooper GM, 2000). The association between specific classes of membrane-binding proteins and the recruitment of lipid species flexible in their shape can lead to membrane deformation and bending (McMahon and Boucrot, 2015; Jarsch et al., 2016; Day and Stachowiak, 2020; Kozlov and Taraska, 2022). Therefore, membrane invaginations, like clathrin-coated pits or caveolae, are formed, and intra- or extracellular membrane vesicles can be constricted from the lipid bilayer, thereby allowing membrane trafficking and stimulation of specific signaling pathways (Kozlov and Taraska, 2022).

In the last few decades, many structural details of the plasma membrane and its physiological function were discovered, such as endocytic structures like clathrin or caveolae, phagocytotic membrane invaginations, lipid rafts, or the formation and release mechanism of exosomes and synaptic vesicles (Cooper GM, 2000; Mayor et al., 2014; Kaksonen and Roux, 2018). Electron microscopy (EM) is primarily used to identify and characterize nanometer-sized structural details of the plasma membrane (Taraska, 2019). Advanced light microscopy, such as total internal reflection fluorescence (TIRF) and super-resolution techniques (stimulated emission depletion microscopy [STED] and STORM), further helped study membrane proteins and their associated physiological processes (Sezgin, 2017; Stone et al., 2017; Chan et al., 2022; Gonschior et al., 2022). Lastly, advancement in structural biology, such as CryoEM and cryo-electron tomography (CryoET), also allows the study of membrane proteins in their (native) lipid environment (Cheng, 2018; Kühlbrandt, 2022).

Metal replicas of membrane sheets enable us to image large areas of the cellular plasma membrane (>500 μm^2^) in high contrast and quantity (Heuser and Kirschner, 1980; Heuser, 2014). To make the inner surface of the basal plasma membrane of adherent cells accessible, the cells are “unroofed” from their cellular body, generating a single plasma membrane sheet attached to the glass coverslip (Heuser and Kirschner, 1980; Heuser, 2000; Svitkina, 2017). Then, a 3–5-nm-thin metal coating of platinum or gold is applied on the plasma membrane sheet. As the handling of metal replicas is delicate, the addition of a carbon coat (ca. 5–8 nm) can help increase the stability of these replicas (Heuser and Kirschner, 1980; Svitkina, 2017). Compared to conventional thin-section EM, the high contrast and topology information gained due to the metal coating is a great advantage to study associated structures, such as actin filaments (Heuser and Kirschner, 1980; Heuser, 2000; Svitkina, 2017; Taraska, 2019). Additionally, large cell areas can be inspected without damaging the biological sample due to long-term exposure to the electron beam. Then, the metal coat prevents heating and, consequently, the damage of the membrane sheets. Platinum replicas of (plasma) membrane sheets, therefore, provide a suitable method for investigating membrane-related processes (Sochacki and Taraska, 2017; Taraska, 2019). However, EM imaging, in general, lacks the ability to provide context regarding protein localization. Immunogold antibody labeling of proteins of interest can help gain insights into specific protein localization in EM samples (D’Amico and Skarmoutsou, 2008; Meier and Beckmann, 2018). Notably, immunogold labeling has limitations compared with light microscopy. Immunogold is notorious either for a very low labeling density or unspecific signal, making it difficult to evaluate exact localization (Bruce et al., 1987). Furthermore, many antibodies cannot recognize the specific antigen after the strong fixation steps used during EM preparation. In contrast, super-resolution fluorescence imaging allows the detection and analysis of proteins by labeling the protein of interest with fluorophores either by endogenous antibody labeling or fluorescent protein tagging (Sezgin, 2017; Stone et al., 2017) at high resolution, allowing the interpretation of protein roles in nanostructures (Schermelleh et al., 2019). Common super-resolution fluorescence techniques include single-molecule localization (resolution limit 10–20 nm, e.g., STORM and PALM), stimulated emission depletion microscopy (STED, resolution limit 40–60 nm), or structure illumination microscopy (SIM and TIRF-SIM, resolution limit ∼110 nm) (Schermelleh et al., 2019; Lelek et al., 2021; Valli et al., 2021; Prakash et al., 2022). Compared to EM techniques, super-resolution fluorescence microscopy reveals detailed information about proteins such as localization, quantity, and their behavior in live cells (temporal and spatial information). However, structural details in Angstrom range cannot be visualized by super-resolution microscopy, although novel approaches combining expansion microscopy and fluorescence fluctuation analysis may overcome this resolution barrier (Shaib et al., 2022). Depending on the super-resolution fluorescence technique used, specific fluorescent dyes, excitation laser (and depletion laser for STED), imaging buffers, and sample preparation protocols must be applied to achieve the highest resolution. Furthermore, sample size and thickness, as well as low labeling density, a high signal-to-noise-ratio, and microscope artefacts (e.g., sample drifting during image acquisition), may diminish resolution barriers (Lambert and Waters, 2017; Valli et al., 2021). Therefore, ideally, the combination of fluorescence microscopy and EM allows the highest spatial resolution and provides the molecular context (Jeong and Kim, 2022).

**FIGURE 1:**
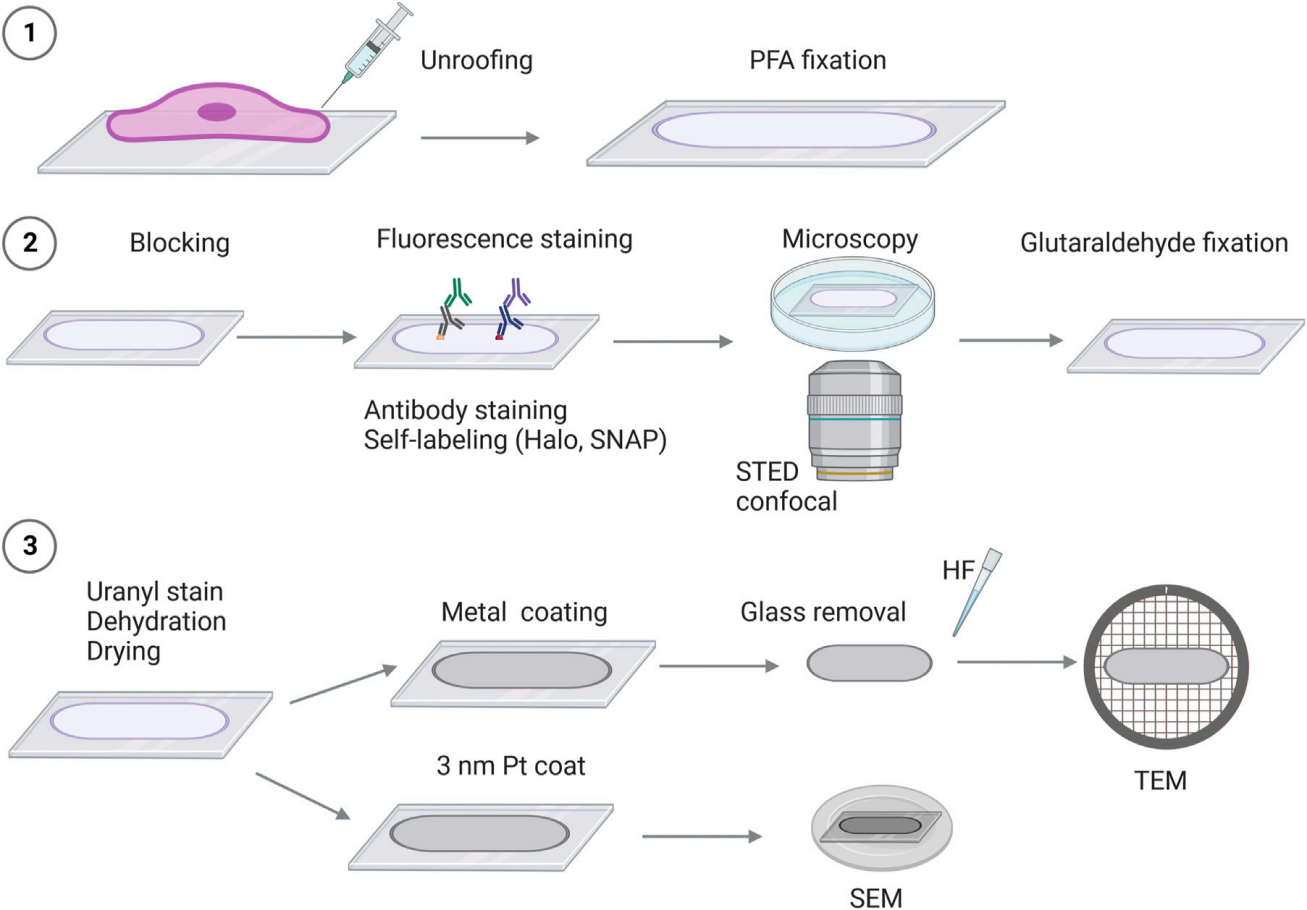
Schematic overview of correlative super-resolution fluorescence imaging and platinum replica electron microscopy (STED-CLEM). 1) Cells are seeded on glass coverslips, followed by the removal of the cellular body (“unroofing”). The adherent plasma membrane sheet is fixed in 4% paraformaldehyde (PFA), followed by staining and/or preparation for fluorescence imaging such as labeling with fluorescent-tagged antibodies or self-labeling of Halo or SNAP-protein tags (2). 2) Proteins of interest are detected by fluorescence microscopy [e.g., stimulated emission depletion microscopy (STED) and confocal microscopy]. After fluorescence imaging, the plasma membrane sheets are fixed in 2% glutaraldehyde (GA). 3) To prepare the membrane sheets for EM, the coverslips are stained with uranyl acetate, and the samples are dehydrated and dried by critical point drying, followed by platinum coating. Depending on the electron microscope used, the glass coverslip will be removed (by HF—hydrofluoric acid), and the platinum replicas can be inspected by TEM. For SEM, the platinum-coated replicas on glass coverslips are mounted on SEM sample holders and can be transferred directly into the SEM microscope for imaging.

Here, we present an adapted correlative super-resolution light and platinum replica EM (CLEM-PREM) approach that enables us to detect specific protein localization to the plasma membrane and can simultaneously assess the underlying membrane and cytoskeleton structures. Previous CLEM-PREM approaches mainly used single-molecule/STORM imaging in combination with platinum replica TEM (Sochacki et al., 2017, 2014; Sochacki and Taraska, 2017; Vassilopoulos et al., 2019; Lemerle et al., 2023). In this paper, we provide a PREM imaging approach combined with STED fluorescence microscopy, making it more applicable for analyzing several targets in the same sample. Additionally, we present an alternative CLEM approach utilizing scanning electron microscopy (SEM) of platinum replicas directly at coverslips, which simplifies and speeds up sample preparation and navigation, allowing much larger areas to be imaged and correlated (Figure 1).

## 2 Methods

### 2.1 Materials and equipment

#### 2.1.1 Cell culture

- Mouse embryonic fibroblasts or cells (cell line) of interest (e.g., ATCC)
- Cell culture medium and appropriate cell culture flasks (DMEM, Gibco #11960044)
- 0.25% Trypsin/EDTA solution (Gibco #25300062)
- D-PBS (Gibco #14190144)
- Plasmid–DNA coding for the EGFP–fusion protein (3–5 µg/6-well, e.g., Addgene)
- Transfection reagent (e.g., Lipofectamine 3000, Thermo Fisher Scientific #L3000001)
- Fibronectin solution (1 mg/mL, diluted 1:100 in PBS, Sigma #F1141)
- Round 25-mm high-precision glass coverslips (#1.5H) with an etched grid (Bellco Biotechnology #1916-91025)

#### 2.1.2 Plasma membrane sheet preparation

- 4% Paraformaldehyde (PFA, EM grade, EM Science #15710, diluted from 16% stock solution in PBS)
- 2% Glutaraldehyde (EM grade, EM Science #16019)
- Plastic syringe with a 19-gauge needle
- 6-well plates
- Stabilization buffer (70 mM KCl, 30 mM HEPES maintained at pH 7.4 with KOH, 5 mM MgCl_2_, and 3 mM EGTA)

#### 2.1.3 STED immunostaining

- BSA (Merck #BSAV-RO)
- PBS (Gibco #10010023)
- GFP nanobody tagged with Atto647N (ChromoTek #gba-647n-100)
- Antibody of interest, including the secondary antibody tagged with suitable STED dye (e.g., anti-rabbit-IgG–Atto647N from Rockland #611-156-122 or anti-mouse-IgG–AlexaFluor594 from Thermo Fischer Scientific #A-11020)

#### 2.1.4 Platinum replica preparation

- 0.1% Tannic acid (diluted in water, EM Sciences #21700)
- Milli-Q water
- 0.1%–0.2% Uranyl acetate (diluted in water, EM Sciences #22400)
- Ethanol (200 Proof, anhydrous, EM grade, EM Sciences #15055)
- Liquid CO_2_ (connected to a critical point dryer via a filter, ultra-pure)
- 4% Hydrofluoric acid (diluted in water, Thermo Fisher Scientific #223335000)
- TEM grids (Formvar/carbon coated on 75 meshes, Ted Pella #01802-F)
- Standard carbon double-sided tabs (Science Services #MN77825-06)
- 25-mm SEM pin stabs (Plano, #G399F) or 4-inch silicon wafer (#SC4CZp-525)

#### 2.1.5 Essential equipment

- Tweezers with fine tips (0.5–0.25 mm)
- For STED microscopy: a suitable STED microscope with a 775-nm depletion laser (e.g., Leica SP8 gated STED with 100× NA1.4 HC Plan Apo CS2 oil objective) and a sample holder for 25-mm round coverslips (e.g., Attofluor Cell Chambers, Thermo Fisher Scientific #A7816)
- Diamond knife (scriber straight 0.5-mm diameter, EM Sciences #62107-ST)
- EM section holder “Perfect Loop” (EM Sciences #70944)
- Critical point dryer for CO_2_ drying, including a sample holder for round coverslips (e.g., Tousimis Autosamdri-815A #8779B or Leica CPD 3000, sample holder tousimis #8767)
- Metal e-beam evaporator, including a carbon and platinum source, rotating and tilted stage (e.g., Leica ACE600 or ACE900)
- Glow discharger for hydrophilization of TEM grids (Ted Pella PELCO #91000S)
- TEM microscope with SerialEM, 80–120 kV (here, Jeol 1400)
- Alternatively, a SEM microscope (here, Helios 5 CX SEM, Thermo Fisher Scientific).

### 2.2 Detailed stepwise procedure

#### 2.2.1 Cell culture and cell seeding

The cell line of interest is cultivated in an appropriate cell culture medium with necessary supplements and grown under suitable CO_2_ and temperature conditions. Here, we use mouse embryonic fibroblasts (MEFs) cultivated in DMEM supplemented with 10% FBS and 1% penicillin/streptomycin (Matthaeus et al., 2020). Twenty-four hours before cell seeding on glass coverslips (with an etched grid), the coverslips are placed in 6-well plates and coated with the fibronectin solution (10 μg/mL) overnight at 4°C. When 80% confluent is reached, MEFs are detached from the culture flask with trypsin/EDTA solution and centrifuged by 1,000 × g for 4 min, and the cell pellet is resuspended in 1 mL DMEM. A total of 100.000 MEFs are seeded on fibronectin-coated coverslips with 2 mL culture medium. The following day, the MEFs are transfected with 2.5–5 µg EGFP–tagged protein of interest (e.g., Cavin1–EGFP) using Lipofectamine3000, according to the manufacturer’s protocol. After 24–48 h incubation, cells are investigated for correct EGFP expression, followed by unroofing and staining. Here, we transfected 2.5 µg Cavin1–EGFP per well and incubated them for 48 h before the cells are unroofed.

Note:

(1) Flat and elongated cells are favorable because unroofing and EM preparation work well for these cells. Cells with a large volume and round shape (e.g., adipocytes) are more difficult to unroof and image. Adhesion of cells can be promoted with different coating reagents, such as fibronectin, collagen, or poly-L-lysin, depending on the cell type.
(2) Transfection with plasmids should be done, as tested beforehand. The plasmid amount, transfection protocol, and incubation time should be adapted as needed.
(3) Phenol red in the cell culture medium can impair fluorescence imaging and increase background autofluorescence. If this is the case, cell culture medium without phenol red should be used for cell cultivation and seeding.

#### 2.2.2 Preparation of plasma membrane sheets (cell unroofing)

Plasma membrane sheets are prepared in a new 6-well plate containing two wells with 2 mL stabilization buffer each and one well with 2 mL freshly prepared 4% PFA for fixation. The cells are unroofed using a syringe with a 19-gauge needle filled with 2 mL 4% PFA.

First, the coverslip with adherent cells is washed in the first well containing the stabilization buffer, followed by transfer to the second stabilization buffer well. Next, the syringe pre-filled with 4% PFA is used to take up the stabilization buffer in well 2 (which contains the coverslip), resulting in a 2% PFA–buffer solution mixture within the syringe (shown in Supplementary Figure S1A). Now, the syringe is moved quickly several times over the coverslip while pressing out the PFA–buffer solution, thereby uncovering cell bodies and forming plasma membrane sheets (Supplementary Figure S1B). Afterwards, the coverslip with the membrane sheets is placed in the third well containing 4% PFA for 15 min.

Note:

(1) Syringes with needles should be tested beforehand. In our experience, 10-mL plastic syringes work the best for generating enough pressure to unroof cells. Furthermore, different needle gauges should be tested for cells of interest. Smaller needle diameters generate a higher pressure on the cells.
(2) Cell types differ tremendously in necessary pressure of the PFA–buffer solution for correct unroofing. Here, it is useful to test several unroofing regimes from “soft” to “harsh” pressure of the PFA–buffer solution to ensure an optimal unroofing process. A conventional membrane stain can be used to inspect plasma membrane sheets immediately after PFA fixation by fluorescence microscopy. If needed, cells should also be seeded on different coating reagents to improve cellular adherence during unroofing.
(3) Notably, unroofing of adherent cells can also be done by sonication. Here, it is helpful to use probe sonicators with microtips, as used for the lysis of small-volume bacterial suspensions. Therefore, the sonicator tip should be placed directly in the stabilization buffer ca. 5–10 mm above the coverslips with the cells (directly in the 6-well plate). Amplitude, pulse mode, and sonication duration should be tested to achieve the most suitable sonicator protocols for unroofing.
(4) Cells should not be stored for long (>1 min) in the stabilization buffer because the buffer is mildly hypo-osmotic, leading to cell swelling. In particular, cell types highly sensitive to osmotic swelling should be handled with caution.

#### 2.2.3 Immunostaining for STED microscopy

After PFA fixation, unroofed cells are washed three times with 1 mL PBS and blocked for 1 h with 3% BSA/PBS. After blocking, antibody staining can be applied. To achieve the lowest label size and highest STED depletion potential, the EGFP–fusion protein of interest is labeled with an anti-GFP nanobody tagged with the fluorescence dye Atto647N. Additionally, we recommend clathrin antibody labeling, which later can be used as an internal correlation marker. Therefore, first, we applied an anti-clathrin heavy-chain antibody (Thermo Fisher Scientific, #MA1-065) diluted to 1:2,000 in 3% BSA/PBS for 1 h at room temperature. Next, coverslips are washed three times with PBS, followed by secondary antibody labeling with anti-mouse-Alexa594 and GFP–nanobody–Atto647N, both diluted to 1:500 in 3% BSA/PBS, for 1 h. Optionally, actin staining, such as Phalloidin–AlexaFluor488 or plasma membrane staining with CellMask dyes (e.g., Thermo Fisher Scientific, #C10046), can be applied for 15 min to reveal unroofed plasma membrane areas during confocal and/or STED imaging. After washing the coverslips three times with PBS, the stained plasma membrane sheets can be stored at 4°C for several days (covered, in the dark) until STED imaging.

Note:

(1) Antibody staining and protein expression levels should be tested to obtain optimal STED imaging results. Suitable STED fluorescence dyes should be used for optimal protein labeling and STED depletion.
(2) High-quality STED imaging can also be done using self-labeling tags, like Halo and SNAP, which allow straightforward labeling with bright and photostable dyes (Bottanelli et al., 2016; Erdmann et al., 2019). This would additionally simplify the protocol because it avoids the immunostaining process.
(3) Clathrin works very well as an internal reference; however, other structural components can also be used, such as actin or caveolae. Notably, a suitable internal correlation structure should be easily detected by antibody staining in STED microscopy (no unspecific antibody labeling) and by its structural appearance in EM (such as the characteristic clathrin lattices; Figure 3B).
(4) Gold fiducials cannot be used for the correlation of fluorescence and EM images because the gold particles strongly heat up the sample when the 775-nm STED depletion laser is used.

#### 2.2.4 STED microscopy

**FIGURE 2:**
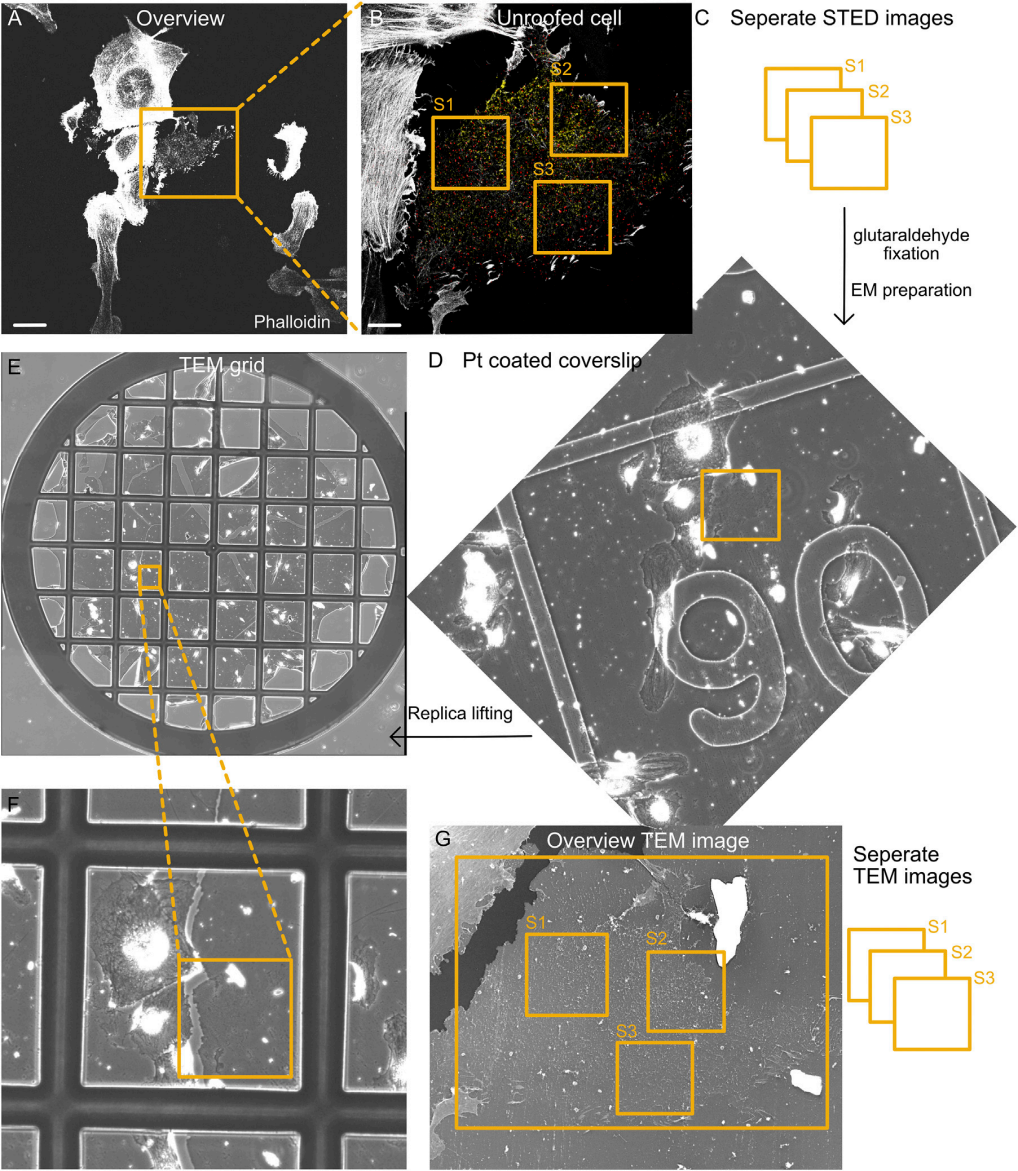
Correlation of fluorescence and EM images. **(A)** Representative tile section of the overview fluorescence image showing intact and unroofed cells labeled by phalloidin (actin). Scale bar: 10 µm. **(B)** Confocal image of the enlarged unroofed cell region from **(A)**. Notably, the unroofed regions can be detected by limited actin staining and a single focal plane that is associated with the plasma membrane. Individual STED images are acquired from different areas of the unroofed cell region (S1–S3). Scale bar: 2 µm. **(C)** STED imaging strategy to investigate several unroofed cell regions per cell (S1–S3). Directly after STED imaging, the location of all imaged cells on the coverslip is identified by the etched grid. Afterwards, the cells are fixed with 2% glutaraldehyde and prepared for EM. **(D)** Part of the overview map obtained by phase contrast microscopy showing the cell region from **(A)** and **(B)** after platinum coating. The etched grid in the coverslip can be easily detected and helps identify the correct area on the coverslip. **(E,F)** After the glass coverslip is removed and replicas are lifted on TEM grids, an overview phase contrast map helps identify the correct cell region. **(G)** TEM image shows the unroofed cell from **(B)**. Again, individual TEM images (e.g., 8 × 8 montages) with a pixel size of 1.2–1.5 nm are acquired that afterwards can be correlated with individual STED images.

STED imaging is performed using a Leica SP8 gated STED microscope equipped with a 100× NA1.4 HC Plan Apo CS2 oil objective and an automatic programmable objective stage, allowing mapping (tile scanning) of large sample areas. Before imaging, coverslips with antibody-stained unroofed cells are washed twice with PBS. Next, coverslips are transferred to a suitable imaging chamber, and 1–2 mL fresh PBS is added. The plasma membrane that remains after unroofing is few nm thick and appears very clearly in confocal images, in contrast to much thicker intact cells. The EGFP signal in the plasma membrane sheets is used to find the correct imaging plane. Next, a region on the coverslip is picked that contains many unroofed cell regions positive for EGFP expression in proximity (4–5 EGFP-positive unroofed cell areas are optimal). Unroofed cell regions can be detected by a clear focal plane (similar to a TIRF imaging plane) without much unfocused (blurry) fluorescence signals. An overview confocal image is taken from the unroofed cell of interest depicting the cell edge and overall cell shape (Figures 2A, B). Next, multiple high-resolution STED images are acquired from the cell of interest (Figures 2B, C). Depending on the STED microscope, STED depletion, sequential imaging of multiple channels, pixel and image size, and signal averaging or accumulation should be optimized for each protein of interest, cell type, or STED dye and, therefore, determine the field of view, resolution, and signal-to-noise-ratio. Here, we use a pixel size of 18.94 nm, 20%–50% STED 775-nm laser depletion power (3–7 MW cm^−2^), and 1%–20% excitation laser power (0.7–17 kW cm^−2^), depending on the fluorescence signal intensity. As shown previously (Matthaeus et al., 2019; Gonschior et al., 2022; Matthaeus C. et al., 2022), we routinely achieve a resolution of ∼50 nm STED. Notably, at least one cell edge should be clearly visible in the STED image for correct correlation with the EM image later.

After high-resolution imaging of cell #1, a large map of the cell region surrounding this cell is acquired (Figure 2A). Importantly, the microscope objective should not be changed. Optimal tile scanning allows the acquisition of 10 × 10 confocal images, generating a large map (883 × 883 μm^2^) with cell #1 in the image center (Supplementary Figure S2A). Tile scans can be acquired with all fluorescence channels or actin alone, making it easy to identify individual cells by their overall shape. This overview cell map will later help identify the cells imaged by STED microscopy and subsequently used for platinum replica preparation.

After generating the overview cell map, the next unroofed cell positive for the EGFP–fusion protein is targeted, and a confocal overview image is acquired, followed by several high-resolution STED images, allowing the correlation and analysis of multiple cell regions (Figures 2B, C, S1–S3). After successful STED imaging of 4–5 different cells, transillumination light is used to identify the etched grid on the coverslip, and the appropriate grid numbers are assigned to the overview cell map (Supplementary Figure S2A). When the coverslip sample holder is removed from the microscope, the residual oil from the objective on the bottom of the coverslip indicates the overall imaging position on the coverslip. We find it useful to mark this on the lower side of the coverslip (using a permanent marker and diamond knife; Supplementary Figures S4B, C). Before placing the coverslip in 2% glutaraldehyde, leftover oil must be carefully removed. Coverslips can be placed in 2% glutaraldehyde for several days at 4°C but minimum for 20 min.

Note:

(1) STED depletion must be tested carefully because with increasing depletion laser power, the membrane and protein structure can be damaged (Kilian et al., 2018; Jahr et al., 2020). Depending on the necessary resolution limits for the visualization of the protein of interest, depletion lasers can be adapted. We recommend using a 775-nm depletion laser (with suitable Atto647N fluorescence STED dye) for targets requiring the highest resolution (∼40 nm, depletion laser powers between 20% and 50% corresponding to 3–7 MW cm^−2^).
(2) Optimal STED resolution and image quality depend on the sample, labeling, and image acquisition, and should be tested beforehand. Image acquisition parameters we used for caveolae proteins are as follows: pixel size 18.94 nm, image size 1,024 × 1,024 pixels, 8 bits per pixel, line average 1 and line accumulation 2 for 647/775 STED laser, 20%–50% STED 775-nm laser depletion power (3–7 MW cm^−2^), 1%–20% excitation laser power (0.7–17 kW cm^−2^), and a scan speed of 600.
(3) Generating an overview map of the cell areas of interest and identifying the underlying grid numbers (etched in the coverslip) are essential for finding the imaged cells during EM preparation steps (Figures 2F, G). All cells imaged by STED should be located within this map (Supplementary Figure S2A).

#### 2.2.5 Platinum replica preparation for TEM or SEM

The coverslips are transferred from glutaraldehyde to PBS and washed three times. Next, tannic acid solution is applied for 20 min, and 6-well plates with coverslips are covered to prevent exposure to light and incubated with tannic acid at 4°C. Afterward, the coverslips are washed ×3 with water, followed by uranyl acetate staining. Again, the 6-well plate with coverslips is placed at 4°C for 20 min. Uranyl acetate is removed in a separate collection bottle, and the coverslips are washed ×3 with water.

Before the unroofed cells on the coverslips can be dried, water is exchanged with ethanol. Therefore, the coverslips are placed in an ethanol dilution row including concentrations of 15%, 30%, 50%, 70%, 80%, 90%, and 100% ethanol. The coverslips are incubated for 4 min in every ethanol dilution step. The final incubation of 100% ethanol is repeated three times. Importantly, the border of the marked region on the coverslip that was imaged in STED (if labeled using a permanent marker) should be scratched using a diamond knife before the coverslips are transferred into ethanol to ensure the relocation of the area of interest (Supplementary Figures S3B, C).

The plasma membrane sheets are dried in a critical point dryer (CPD), whereby ethanol is exchanged with liquid carbon dioxide (CO_2_), followed by an appropriate temperature and pressure increase to exceed the critical point for CO_2_ (30.98°C and 73.75 bar). Afterward, the pressure is reduced, allowing the transition from the CO_2_ critical phase to the gaseous phase. Depending on CPD, the automatic heat–pressure program must be adjusted according to the manufacturer’s recommendations, allowing us to reach the critical phase of CO_2_. After gaseous CO_2_ is removed, the coverslips with dried plasma membrane sheets can be inspected, and the diamond knife-scratched regions are removed for metal coating.

The coverslip pieces with the dried plasma membrane sheets are stuck to a double-faced adhesive tape, placed on an appropriate sample holder, and transferred into a metal e-beam coater (e.g., Leica EM ACE900 or 600; Supplementary Figure S3D). After high vacuum (<10^−6^ bar) is established, the sample stage is tilted to 17° and rotation is started (ca. 20 rpm). First, a 3-nm platinum coat is applied, followed by a 5.5-nm carbon coat (see illustration in Supplementary Figure S4). After the coverslips are removed from the sample holder, the coated (unroofed) cells can be inspected by transillumination light with a 40× objective. The grid should be easily detected and helps identify the region of interest (Figure 2D). Based on the overview map (acquired during STED imaging) and the etched grid on the coverslip, the cells imaged by STED microscopy can be identified, and a phase contrast image of this area is acquired. Additionally, we find it useful to mark the distinct areas with imaged cells on the coverslip directly at the microscope using a permanent marker. The following procedure of this section describes processing of the samples for TEM imaging. If the coated membrane sheets are investigated by SEM instead of TEM, the procedure continues with that explained in Section 2.2.7. For TEM imaging, the coverslip needs to be trimmed to fit the size of TEM grids. This is achieved by cutting the coverslip using a diamond knife into small pieces, with the size of TEM grids being approximately 3 mm × 3 mm.

Before placing the replicas on the TEM grids, the coated membrane sheets must be separated from the glass coverslip. Therefore, the cut coverslips are placed in 1 mL hydrofluoric acid with glass below and the platinum and carbon-coated side looking up (in a 12-well plate). Based on their low weight and the surface tension of the diluted hydrofluoric acid, the replica floats in the solution. After 2–4 min, the glass dissolves from the Pt replica and sinks to the well bottom. Now, the floating replica is washed with 30–40 mL water by repetitively and carefully pipetting water in and out of the well. The washed replicas are collected with the “perfect loop” (for lifting EM sections) and placed on TEM grids on a filter paper. The EM grids should be dried for at least 10 min before they are placed in grid boxes or imaged by TEM.

Note:

(1) Uranyl acetate is radioactive and should be handled with caution and in line with appropriate local regulations. We find it useful to cover our hands with two pair of gloves. Afterwards, all plasticware that handled the uranyl acetate solution should be disposed in designated containers. We prepare a 2% uranyl acetate stock solution that can be stored at 4°C for several months.
(2) Hydrofluoric acid is highly corrosive and can penetrate organic tissues rapidly (highly toxic). It should be handled with caution, only under the fume hood, with appropriate safety measurements (including gloves, glasses, and laboratory coat).
(3) To ensure optimal EM imaging results, correct drying of the membrane sheets is essential. Therefore, it should be observed in CPD if the critical point is reached within the sample chamber. If the critical point is not reached, the dried membrane appears squeezed or collapsed because of high pressure on the sample.
(4) After critical point drying, the coverslips should be metal-coated immediately because air humidity can harm dried plasma membrane sheets.
(5) When cutting Pt-coated coverslips regularly, several coverslip pieces are generated with different cells of interest. All coverslip pieces should be investigated by EM to ensure the relocation of unroofed cell areas investigated by STED. We find it useful to stick the Pt-coated coverslips to a double-faced adhesive tape (the metal side showing up) and place them on a small plastic dish (e.g., 3-cm round cell culture dish). Therefore, during cutting, the coverslip pieces cannot move around and will stick to the adhesive tape.
(6) TEM grids should be hydrophilized using a glow discharge system shortly before use for metal replica placement (maximum 30–60 min before use).

#### 2.2.6 TEM imaging

Before the EM grids are investigated by TEM, we recommend briefly inspecting the grids by transillumination light microscopy with a 40× objective. By comparing phase contrast images of the regions of interest in platinum-coated coverslips (before coverslip removal) to EM grids, the cells imaged by STED can be found again (Figures 2E, F).

**FIGURE 3:**
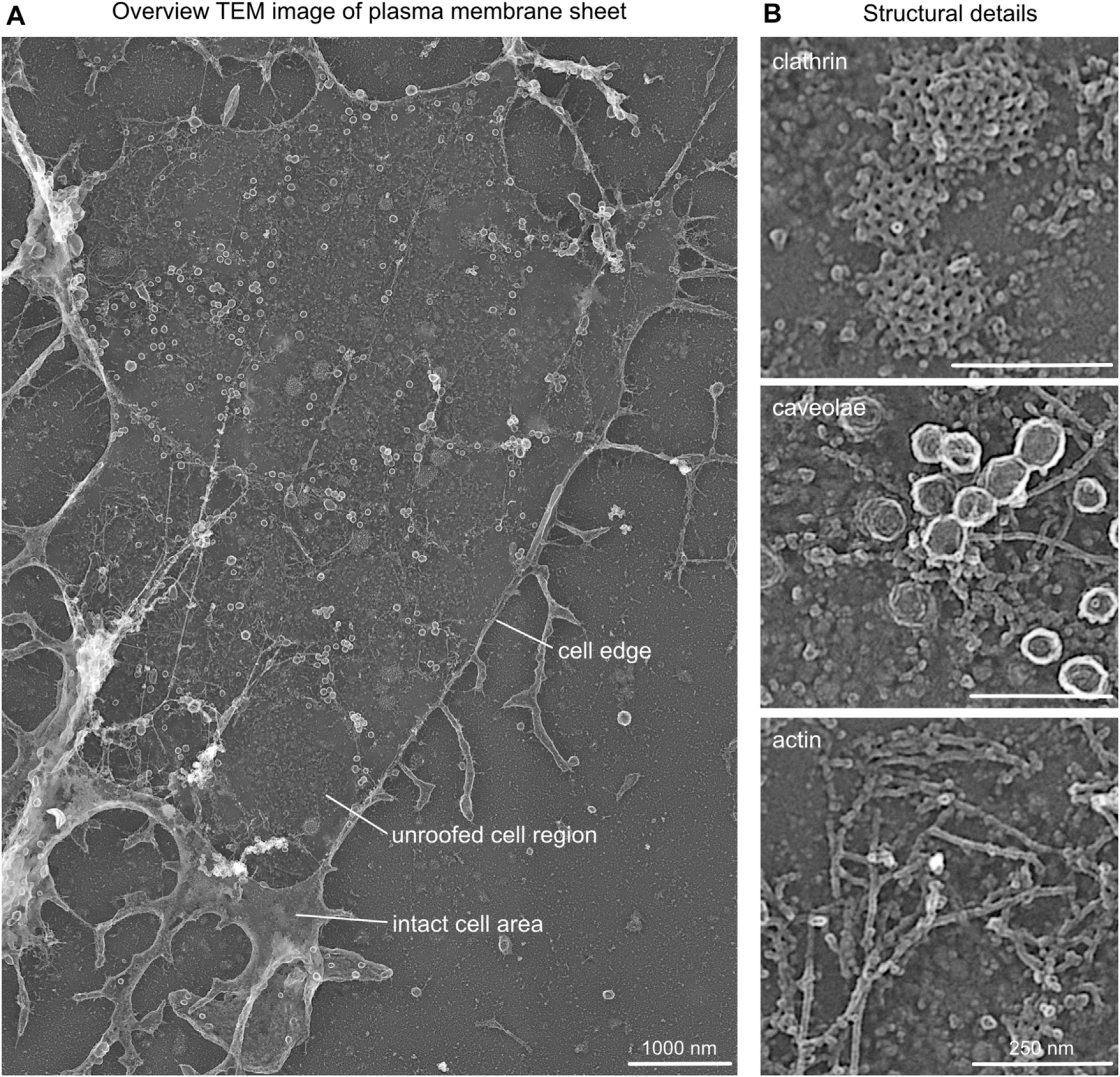
Structural details in platinum replica TEM images. **(A)** Representative overview TEM image showing a MEF plasma membrane sheet. Cell edge, unroofed plasma membrane region, and intact apical cell area are highlighted. **(B)** Characteristic structural components of plasma membrane sheets that can be used as internal correlation markers include clathrin lattice, caveolae, and actin filaments.

As the platinum replicas show large areas of plasma membrane sheets, it is useful to image the grids in TEMs containing an automatically movable stage, allowing us to acquire large tile scans (e.g.,: 8 × 8 images, 64 images in total; Figure 3A). We find SerialEM (Mastronarde, 2005) useful to operate and allocate the desired cell regions. In general, we use pixel sizes of 1.2–1.8 nm and exposure times between 2 and 6 s, and operate the microscope at 120 kV (Jeol 1400 TEM). Tile scan images are reconstructed by IMOD (Kremer et al., 1996). Cell regions imaged previously by STED microscopy can now be inspected by TEM.

Note:

(1) Tile scans should be acquired with 10%–20% overlap of neighboring images to allow optimal reconstruction.
(2) When imaging regions of interest, the TEM image should contain cell borders (accordingly to the STED image) for a better correlation of STED to the TEM image (Figure 3A).
(3) The platinum coat of (unroofed) cells protects samples from electron beam etching due to high electron densities. However, the Formvar coat on EM grids can be damaged by longer exposure times.

#### 2.2.7 SEM imaging

For SEM imaging, coverslips that underwent critical point drying and low-angle coating with platinum and carbon are attached to any SEM holder of choice (e.g., 25-mm pin stab) with a double-sided conductive carbon tab. To identify the cell region of interest, low-resolution scanning of the coverslip with an ICE or ETD detector is performed to generate an overview image for navigation. Regions of interest can then be correlated to the confocal overview image taken during light microscopy using MAPs software (Thermo Fisher Scientific). For high-resolution SEM imaging, the immersion mode is used, and scanning is performed with a pixel size of 0.674 nm without any significant damage to the sample. Optimal imaging conditions are obtained with an electron beam with a current strength of 11–21 pA at 5–10 kV. Secondary electrons are detected using a through-the-lens detector (TLD) at a dwelling time of 1–2 ɥ and 4–8 times line integration to negate potential charging. In case gold fiducial markers were used, multidetector imaging can be used, parallel to TLD secondary electron detection, and a mirror detector (MD) could be activated, which will preferentially detect backscattered electrons. Thus, gold particles will be visible in the MD channel (Supplementary Figure S5). Once SEM imaging is performed, proceeding to TEM is still possible. To do so, coverslips can be carefully detached from the SEM holder using a razorblade, and coverslip trimming and replica lifting from the glass coverslip can be done, as described in Section 2.2.5.

Note:

(1) Standard coating conditions for plasma membrane sheets of 3-nm platinum and 5.5-nm carbon provide sufficient conductivity for SEM imaging. However, structures appear less fine as compared to TEM due to carbon coating, which is not visible in TEM (Figure 5). How much carbon coating can be reduced for purely SEM probes, without reducing the scanning quality due to charging, depends on the sample and must be determined beforehand.
(2) Navigation in SEM, especially if coverslips with etched markings are used, is very easy. Thus, whole coverslips can be mounted.
(3) In case multichannel imaging is used for simultaneous fiducial detection, 10 kV might provide a better BSE signal from gold particles. If only secondary electrons are detected, imaging at 5 kV provides better images.
(4) We noted no obvious electron damage of the replica, following SEM imaging, notable in TEM, so SEM prior to TEM, *per se*, has no negative effect. The only issue one has to be very careful while detaching the coverslip from the SEM pin or wafer using a one-sided razor is to not break the glass itself. Once dispatched, the glass coverslip with ROI is cut using a diamond knife into 3–4-mm pieces for replica lifting (2.2.5).

#### 2.2.8 Correlation of the STED fluorescence image with the EM image (CLEM)

**FIGURE 4:**
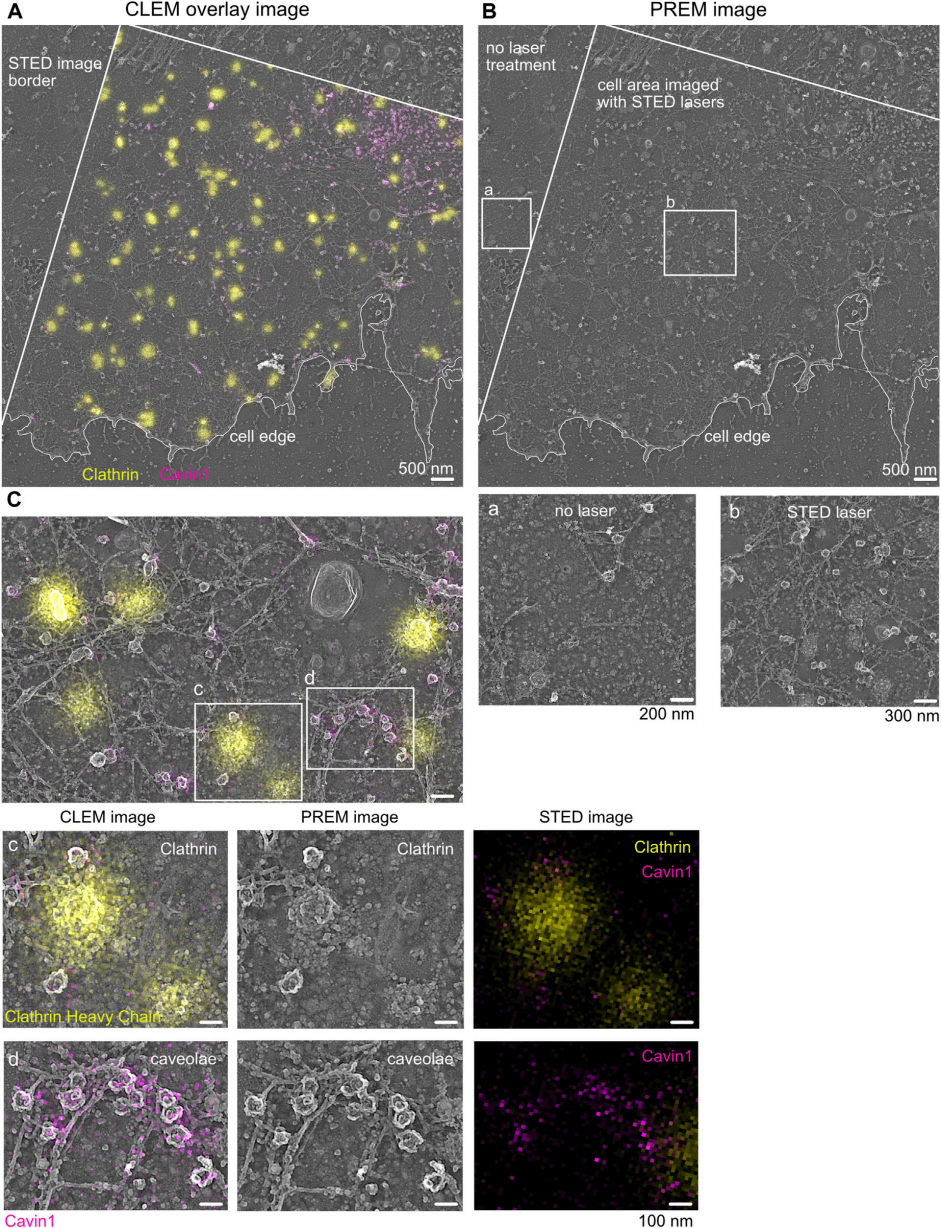
Correlative STED and Pt replica TEM image (CLEM). **(A)** Representative STED-PREM overview image of the MEF plasma membrane sheet. STED image border and cell edge are indicated in white; clathrin (yellow) was immunolabeled by a specific antibody and used as an internal correlation marker, and caveolae were tagged by Cavin1–EGFP (magenta). Scale bar: 500 nm. **(B)** PREM image [from **(A)**] showing the membrane sheet that was imaged by STED microscopy compared to membrane areas (zoom box b) not exposed to STED excitation and depletion lasers (zoom box a). Scale bar: 500 nm. **(C)** Enlarged plasma membrane region from **(A)**. Zoom box c indicates clathrin lattices excessively labeled with the clathrin antibody (yellow). Zoom box d shows caveolae and the associated Cavin1–EGFP signal (magenta). Scale bar: 100 nm.

Before the alignment of STED and EM images, the TEM images are inverted using Fiji/ImageJ (Rueden et al., 2017) (white to black) in order to obtain a SEM-like appearance, allowing an intuitive and detailed inspection of the plasma membrane sheets. Next, separate images of each fluorescence channel (e.g., 647 and 594 nm) in the corresponding STED images are generated. For correlation, we recommend either Fiji plugin BigWarp (IEEE Engineering in Medicine and Biology Society, 2016; Russell et al., 2017) or a MATLAB code published previously (Prasai et al., 2021; Matthaeus C. et al., 2022). First, coarse alignment can be carried out using confocal images of actin or plasma membrane staining. Here, the cell borders detectable in fluorescence and EM images are used as a coarse correlation marker, helping to overlay both images. Next, correlation is refined within the plasma membrane sheet by associating distinct clathrin structures (in the EM image) with their specific fluorescence signal in the STED image. After successful correlation, a STED–EM merge image (CLEM) is generated, and STED fluorescence can be inspected in detail within the EM images (Figures 4, 6).

Note:

(1) During the preparation of the membrane sheets, minimal movement and distortions of lipids, membrane domains, actin filaments, or similar can occur, causing an inefficient correlation of fluorescence images to EM images in some areas (Supplementary Figure S6). We recommend improving the correlation using markers within the cellular area of interest, while cell borders or large floppy membrane areas should be avoided for the alignment.
(2) Depending on the correlation program used, multiple STED fluorescence channels can be correlated with the individual EM image in one correlation step. Otherwise, for each STED fluorescence image, the coarse and fine correlations (cell border and internal marker) must be repeated separately.
(3) Gold fiducials can be used for alignment when confocal or PALM/STORM imaging is performed. Gold fiducials, however, cannot be used for the correlation of STED fluorescence and EM images because the gold particles strongly heat up the sample when the STED depletion laser is used.
(4) External fiducials are also not 100% reliable; not all retain fluorescence, location, or structure. Fiducials or structures can move during the sample preparation step. Thus, in practice, majority of CLEM experiments commonly use internal and, preferably, local fiducials for the correlation of fine structures (nanoscale). In our study, clathrin, actin, and plasma membrane stain were ideal internal fiducials to align light and electron microscopy features.

## 3 Results and discussion

### 3.1 STED-TEM correlation

As shown in Figure 4, the overlay of the acquired STED image and its corresponding TEM image indicates the correct correlation of both images. A detailed inspection revealed that clathrin fluorescence is associated with the correct underlying clathrin EM structure (Figure 4C, yellow). Additionally, caveolae correlation of Cavin1 STED fluorescence with its specific EM coat structure was also possible (Figure 4C, magenta). Both plasma membrane structures show detailed characteristics such as shape, coat, and electron contrast in the TEM image, which makes them suitable as an internal correlation marker. A detailed inspection of larger areas of correlated membrane sheets revealed that in some areas, the Cavin1 fluorescence in the STED image was shifted relative to its associated TEM region (Supplementary Figure S6B). This can be caused by drifting or moving of membrane components during or after STED imaging or sample shrinkage during drying (such as the caveolae neck or floppy phagocytic membrane invaginations). To circumvent potential mis-correlated, shifted membrane areas, harsh temperature changes between STED imaging and EM fixation and EM preparation steps (staining and dehydration) should be avoided. Notably, the correlation of plasma membrane components in close relation to the cell edge should be analyzed with caution as the cell edge is more prone to shrinkage and movement during EM preparation.

Notably, increased STED laser intensity can damage the ultrastructure of the plasma membrane. In particular, the membrane lipid bilayer can be impaired by increasing STED depletion laser power, leading to reduced crispiness in the corresponding TEM images. When comparing several STED depletion regimes, we observed in cells imaged with >70% 775 nm STED depletion laser intensity (∼7–9 MW cm^−2^) more damaged membrane areas. Therefore, we recommend testing several STED depletion intensities, followed by a rigorous inspection of the underlying membrane structure and cytoskeleton to establish optimal STED imaging conditions. As shown in Figures 4B, C, appropriate STED depletion laser power helps preserve the ultrastructure of the membrane and allows super-resolution fluorescence imaging.

The correlated STED-TEM images can now be inspected with regard to the protein or structure of interest. Notably, a suitable analysis method must be developed to extract the necessary information. We find it useful to generate cell masks of the overall plasma membrane sheets in which specific structural features are marked, as shown in Supplementary Figure S7 (in Fiji/ImageJ, e.g., caveolae or clathrin). Based on these masks, the nanoscale localization, size, and quantity of plasma membrane structures and associated proteins of interest can be analyzed quantitatively (Supplementary Figure S7E, analysis of the fluorescence profile of Cavin2 proteins over the caveolae coat) (Sochacki et al., 2021; Matthaeus C. et al., 2022). Specifically, it is possible to extract and average structures or measure distances between membrane structures and proteins down to a distance of 40 nm depending on the alignment.

### 3.2 Light microscopy–SEM correlation

**FIGURE 5:**
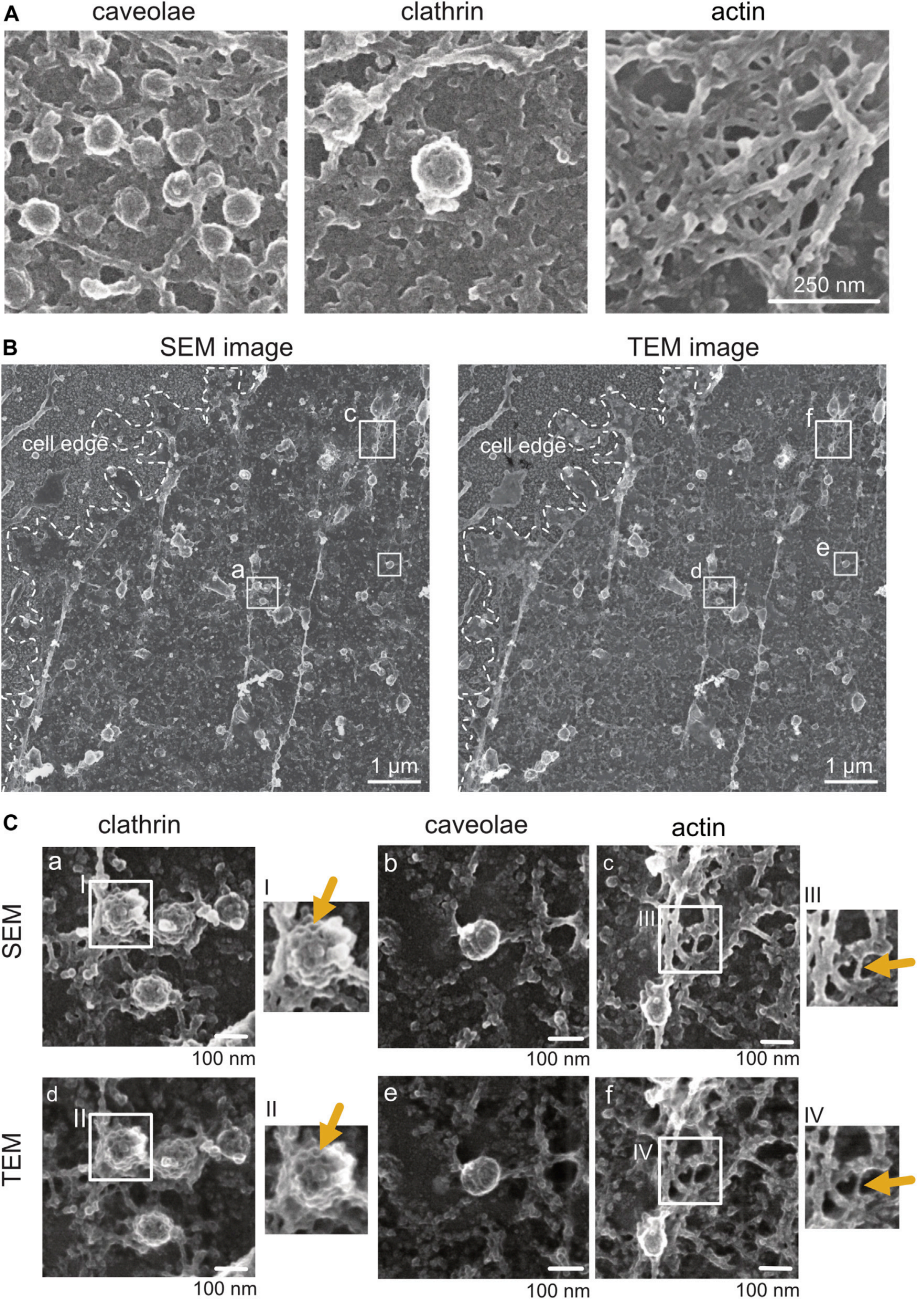
Comparison of platinum replica plasma membrane sheets imaged by SEM and TEM. **(A)** Representative SEM images of caveolae, clathrin, and actin structures from plasma membrane sheets of a NRK49F cell. Secondary electrons were detected using the through-the-lens detector (TLD). Scale bar: 250 nm. **(B)** On the left is an overview image of a Pt replica membrane sheet of an unroofed NRK49F cell imaged first by SEM (TLD SE) at the glass. On the right is the same cell overview after the Pt replica was lifted from the coverslip and transferred onto the grid for TEM inspection. Transmitted electrons (for the TEM image) were detected by the brightfield sector of a retractable STEM detector. Boxed regions of clathrin lattices (a, d), caveolae (b, e), and actin (c, f) are magnified in **(C)**. Scale bar: 1 µm. **(C)** Representative SEM and corresponding TEM images showing structural details of clathrin, caveolae, and actin from the same unroofed NRK49F sample. Arrows point to structures that appear less fine and thin in SEM compared to TEM. Scale bar: 100 nm.

**FIGURE 6:**
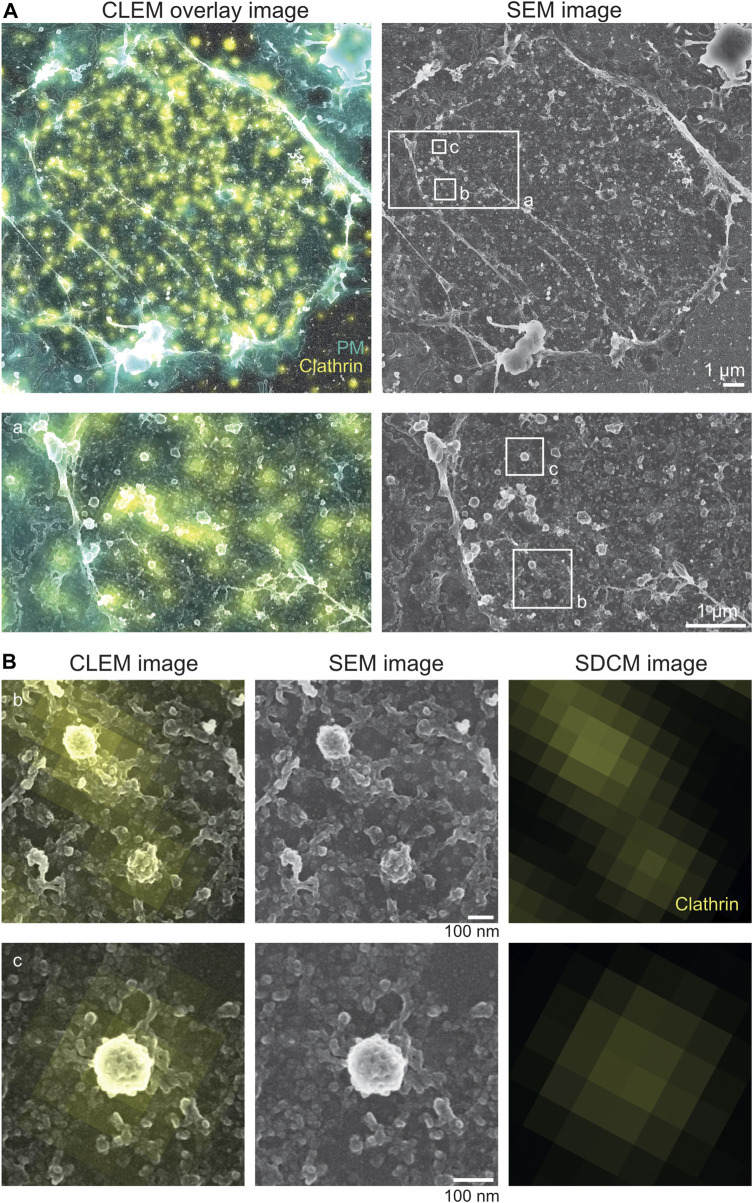
Correlative light microscopy and Pt replica SEM image. **(A)** Representative correlative spinning disc confocal microscopy (SDCM) and platinum replica SEM image of a NRK49F plasma membrane sheet. Overlay of SDCM and SEM image is shown on the left, and the SEM image is shown on the right. The region of white border (a) is magnified thereafter. Clathrin (yellow) immunolabeling and plasma membrane (PM, in cyan) staining were used as internal correlation markers. Scale bar: 1 µm. **(B)** Enlarged plasma membrane regions (b, c) from **(A)**. Magnifications show clathrin lattices excessively labeled with the clathrin antibody (yellow). Scale bar: 100 nm.

The inspection and analysis of light microscopy–SEM images can be done in the exact same way, as mentioned for correlated STED-TEM images (Figure 6). The resolution of modern field emission SEMs is sufficient to identify clathrin structures, actin filaments, and caveolae (Figures 5A, B). For the replica ultrastructure, secondary electrons are detected, and for gold fiducials, if present, backscattered electrons (Figure 5A; Supplementary Figure S5). Although sufficient in identification quality, ultrastructures in SEM appear to be less fine (Figure 5B). In order to confirm this observation, following SEM imaging, we dismounted the coverslip from the SEM holder, removed the replica from the coverslip, and transferred the replica of the membrane sheets onto a TEM grid. Therefore, we compared the same ROI imaged by SEM in the brightfield TEM mode (with a retractable STEM detector of Helios 5 CX) (Figure 5B). Indeed, when inspecting clathrin lattices, the intermolecular space between neighboring clathrin chains appears more filled with electron-dense material in SEM than the empty space in TEM (Figure 5C, comparison between SEM and TEM). The reason for this lies in the type of detected electrons. For TEM, transmitted electrons are disproportionally deflected by the platinum coat, and the 4–6 nm carbon, which was applied for replica preservation, is relatively transparent for electrons at 30–120 kV. In contrast, for SEM, the detected electrons are secondary electrons that stem from the carbon coat. However, since the PREM resolution is generally limited due to the nature of platinum and carbon deposition, practical reduction in resolution is negligible. Thus, SEM offers a number of advantages over TEM, notably, simpler preparation of samples after light microscopy, as well as overall navigation on the sample and the correlation of SEM with light microscopy images with similar end results compared to STED-TEM (Figure 6). Specifically, clathrin and actin can be detected easily, making the STED-SEM approach suitable to various research questions (Figure 6B). Depending on the research aim and structure/protein of interest and tools available, the most suitable method should be applied.

### 3.3 Conclusion and limitations

Combining super-resolution light microscopy and correlative electron microscopy allows us to detect and characterize proteins associated with specific plasma membrane areas in high resolution (De Boer et al., 2015; Jeong and Kim, 2022; van den Dries et al., 2022). Previous studies using platinum replica EM commonly combined PALM/STORM to detect proteins, making this CLEM approach technically very demanding (Sochacki et al., 2017, 2014; Kopek et al., 2017). Here, we present an adapted version of the replica CLEM protocol for studying processes at the plasma membrane including a step-by-step manual with suggested improvements and facilitations. Thus, despite somewhat lower resolution, using STED microscopy significantly increases the throughput due to faster sample preparation and imaging time than in STORM/PALM. Using SEM instead of TEM further simplifies navigation on coverslips and omits challenges associated with successful replica lifting from the underlying glass coverslip and transfer onto the TEM grid (which is necessary for TEM). Resolution difference between SEM and TEM is rather negligible for practical purposes, and specifically, a high-quality critical point drying/platinum coating is responsible for good imaging results. Notably, after SEM, one can still perform classical replica TEM, in case more resolution, contrast, or tomography would be required.

Platinum deposition thickness inherently limits the resolution of ultrastructural details to ∼2–3 nm. However, the combination of platinum replica electron microscopy (TEM or SEM) and super-resolution light microscopy enables us to extract specific information about protein localization, combined with high contrast and resolution achieved by EM. In particular, characterization of plasma membrane processes benefits from this CLEM method. However, by its nature, EM images cannot reveal any temporal details due to its need for cell fixation. Therefore, highly dynamic processes can be difficult to catch and investigate by this CLEM method and may require chemical or optogenetic tethering as well as (high-throughput) enrichment strategies. In addition, the generation of plasma membrane sheets by unroofing cells may lead to the removal of plasma membrane-associated structures due to the applied shear force. Furthermore, weak protein–protein or protein–lipid interactions may be disturbed during unroofing. Depending on the protein of interest, this should be tested beforehand by confocal and/or STED microscopy by comparing structures in live, fixed, and unroofed cells. In addition, harsh STED depletion laser power can destroy the membrane sheets and may lead to artefacts visible in EM images. This should always be tested before the actual CLEM experiment is performed.

In summary, the described correlative super-resolution light and electron microscopy approach will help determine plasma membrane processes and can extract quantitative information based on appropriate analysis techniques. Depending on the target of interest, protein localization at the plasma membrane, membrane bending, curvature status, or actin association can be determined. For example, the localization of specific proteins to caveolae can be evaluated, as well as clathrin pits at specific stages of maturation, cellular adhesion points, particularly dense structures as midbodies, and initial segments of axons. For studying endo- or exocytosis, this method shows great potential to identify molecular details in the membrane remodeling processes. Analysis of cells unroofed by alternative methods, like membrane rip off or detergent-based PM removal, allows the exposure of intracellular surfaces beyond the plasma membrane, which might be another potential application (Yang and Svitkina, 2019). Thus, the cellular interior of the mitochondria, endoplasmic reticulum, lysosomes, and cytoskeletal elements could be also imaged with this method.

## Data Availability

The original contributions presented in the study are included in the article/Supplementary Materials; further inquiries can be directed to the corresponding author. STED-TEM datasets analyzed in this study can be found on figshare: https://figshare.com/collections/The_molecular_organization_of_differentially_curved_caveolae_indiindic_bendable_structural_units_at_the_plasma_membrane_/6253644.
