# Supplementary material for: Analyzing the cellular plasma membrane by fast and efficient correlative STED and platinum replica EM
Source: Front Cell Dev Biol. 2023 Nov 30;11:1305680. doi: 10.3389/fcell.2023.1305680 (PMC10720448; doi:10.3389/fcell.2023.1305680)
Supplement: Supplementary file 1 [file DataSheet1.pdf]

frontiers

Supplementary Material

Analyzing the Cellular Plasma Membrane by Fast and Efficient Correlative STED and Platinum Replica EM

Dmytro Puchkov[1], Paul Markus Müller[2], Martin Lehmann[1], Claudia Matthaeus[3*]

[1] Cellular Imaging Facility, Leibniz-Forschungsinstitut für Molekulare Pharmakologie, Berlin, Germany

[2] Institute for Chemistry and Biochemistry, Freie Universität Berlin, Berlin, Germany

[3] Cellular Physiology of Nutrition, Institute for Nutritional Science, University of Potsdam, Potsdam, Germany

*** Correspondence:**
Claudia.matthaeus@uni-potsdam.de

SUPPLEMENTARY FIGURES :

Figure S1: Schematic overview of unroofing process.

Figure S2: Relocation of unroofed cell regions of interest on coverslips.

Figure S3: Technical details of CLEM workflow.

Figure S4: Schematic illustration of platinum replica.

Figure S5: Gold fiducials in SEM multi detector modality.

Figure S6: Structural details in CLEM images.

Figure S7: Analyzing CLEM images.

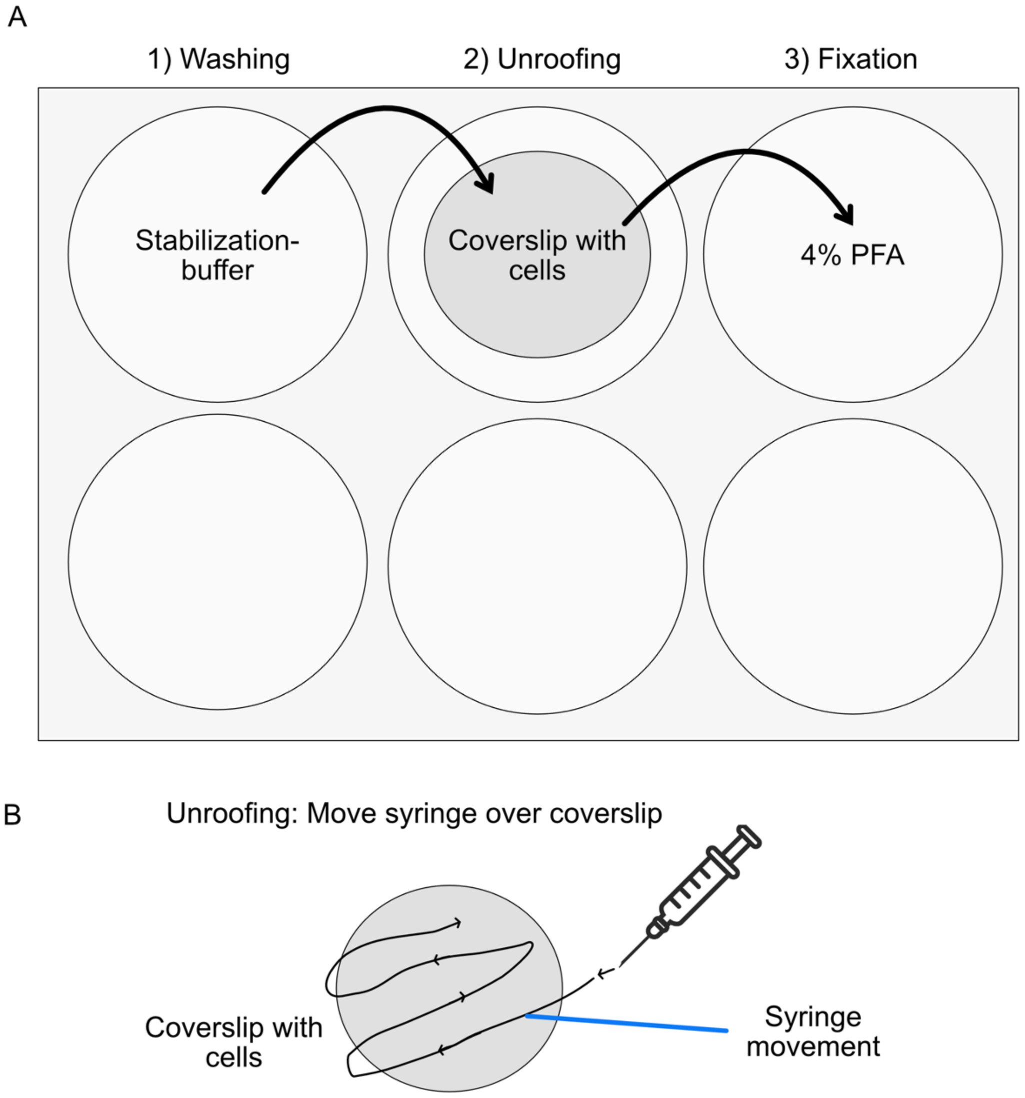

Supplementary Figure S1: Schematic overview of unroofing process.

(A) Cells of interest are seeded on glass coverslips. For preparing plasma membrane sheets the coverslip will be transferred into a 6-well containing stabilization buffer for washing, followed by transfer into second well containing 2 ml stabilization buffer for unroofing with PFA (see B). After unroofing the coverslip is transferred into a new well containing 4 % PFA for fixation.

(B) For unroofing 10 ml plastic syringe (with 19-gauge) is filled with 2 ml 4% PFA. When coverslip is transferred into unroofing 6-well, the 2 ml stabilization buffer is sucked into the syringe resulting in 4 ml 2% PFA-stabilization buffer solution. To unroof cells seeded on the coverslip, the syringe is now emptied with pressure whereby the syringe is moved over the coverslip as illustrated. This movement allows the unroofing of large areas of the coverslip.

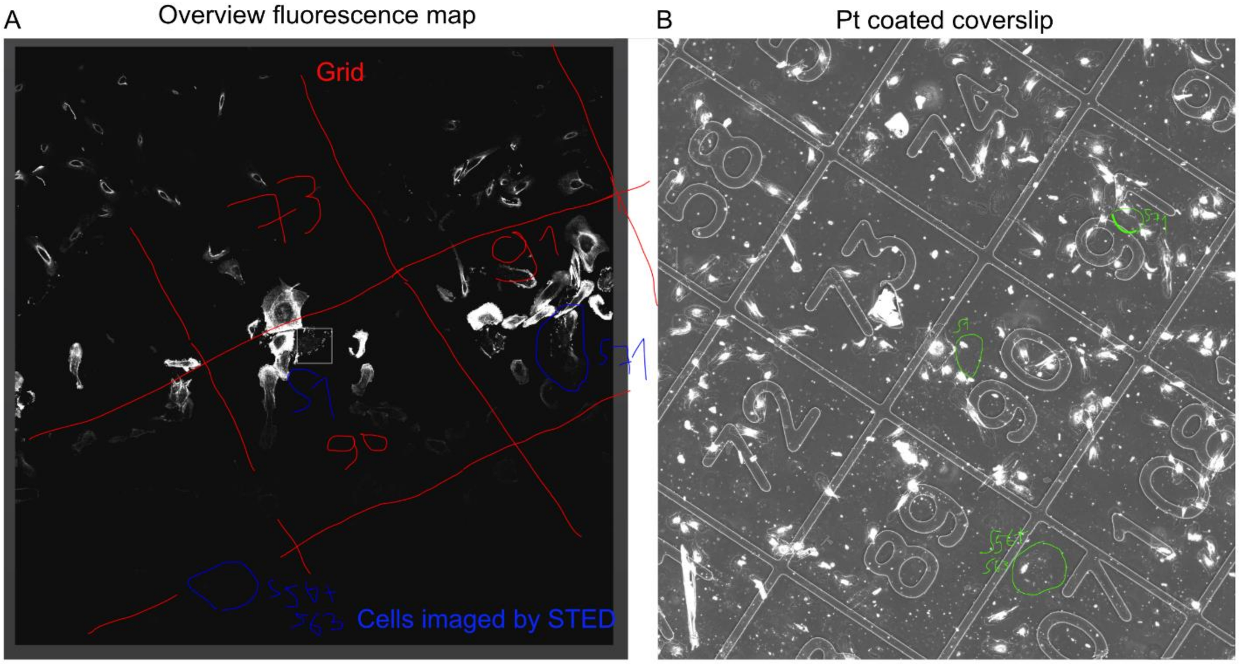

Supplementary Figure S2: Relocation of unroofed cell regions of interest on coverslips.

(A) Confocal overview map (10 x 10, corresponds to fig. 2) with depicted grid boarders and numbers (in red). Grid number and location was determined with transillumination light after STED imaging with the same microscope objective (100x). Unroofed cell regions imaged by STED microscopy are marked in blue. Cells were stained with Phalloidin-Alexa488 to identify cellular shapes and unroofed areas (in white).

(B) Corresponding phase contrast map showing platinum coated coverslip with etched grid and numbers. Relocated cell regions imaged by STED are marked in green. For TEM, coverslips are cut in smaller pieces (ca. 3 mm x 3 mm) allowing to place them on TEM grids.

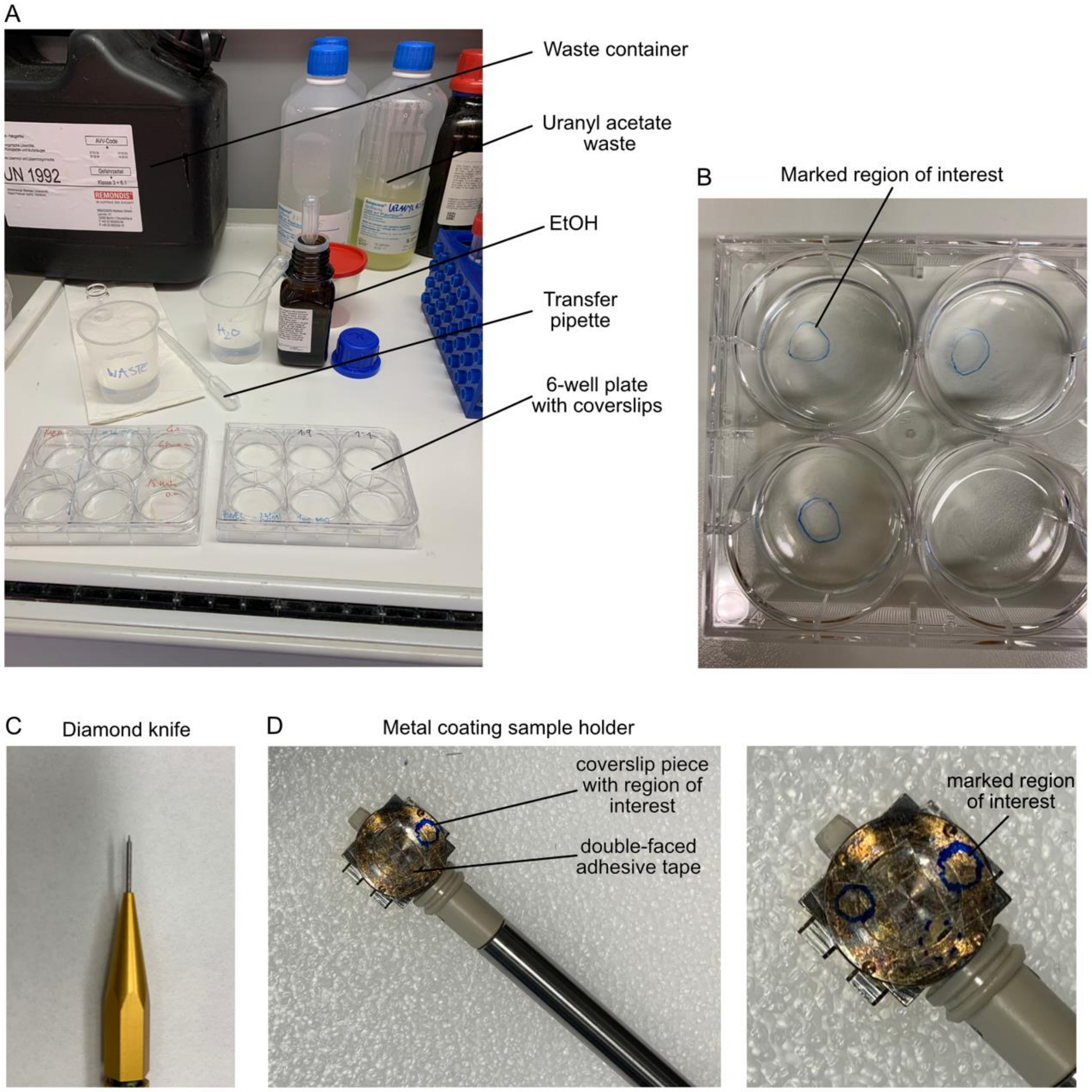

Supplementary Figure S3: Technical details of CLEM workflow.

(A, B) CLEM preparation in chemical hood (A). The region of interest that was imaged by STED microscopy were marked by pen directly on coverslip (B) and transferred to 6-well plate for metal replica preparation.

(C) Diamond knife used to mark and cut region of interest on coverslip.

(D) Coverslip pieces with region of interest are placed on sample holder with double-faced adhesive tape.

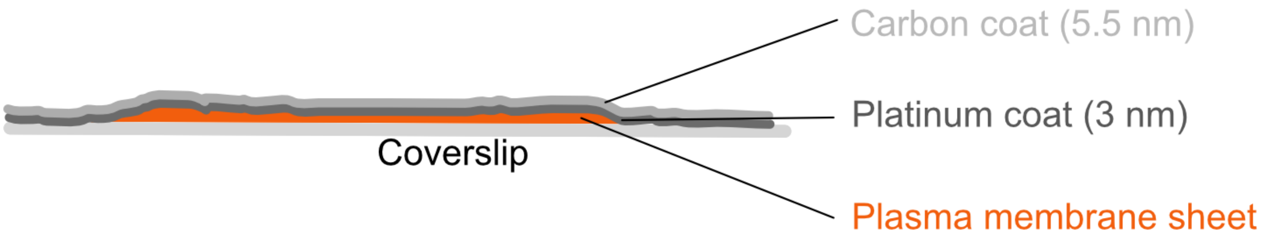

Supplementary Figure S4: Schematic illustration of platinum replica.

Unroofed plasma membrane sheets (in orange) are at first coated with 3 nm platinum (dark grey), followed by 5.5 nm carbon coating (light grey). Afterwards, the platinum replica can be investigated by TEM, however the glass coverslip must be removed with hydrofluoric acid beforehand. In contrast, when imaging platinum replicas by SEM the replica with its underlying glass coverslip can be attached directly to SEM sample holder. Please note the illustration does not represent original scale.

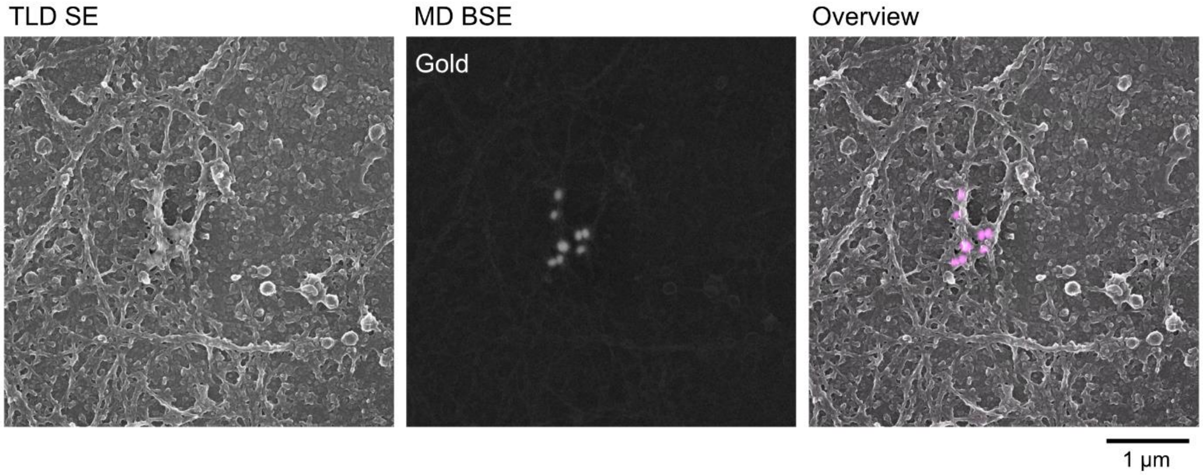

Supplementary Figure S5: Gold fiducials in SEM multidetector modality.

Representative SEM sample including gold fiducials for correct alignment of fluorescence and SEM images by using multidetector SEM imaging. Secondary electron detection (TLD SE) will be applied to image plasma membrane sheets to visualize structural details. Mirror detector back scattered electrons (MD BSE) will be detected to identify gold fiducials. Overview image shows merge of TLD SE and MD BSE detection. Note gold particles (50nm) visible in magenta in overlay image and as white dotes in BSE only image gold particles are marked cyan.

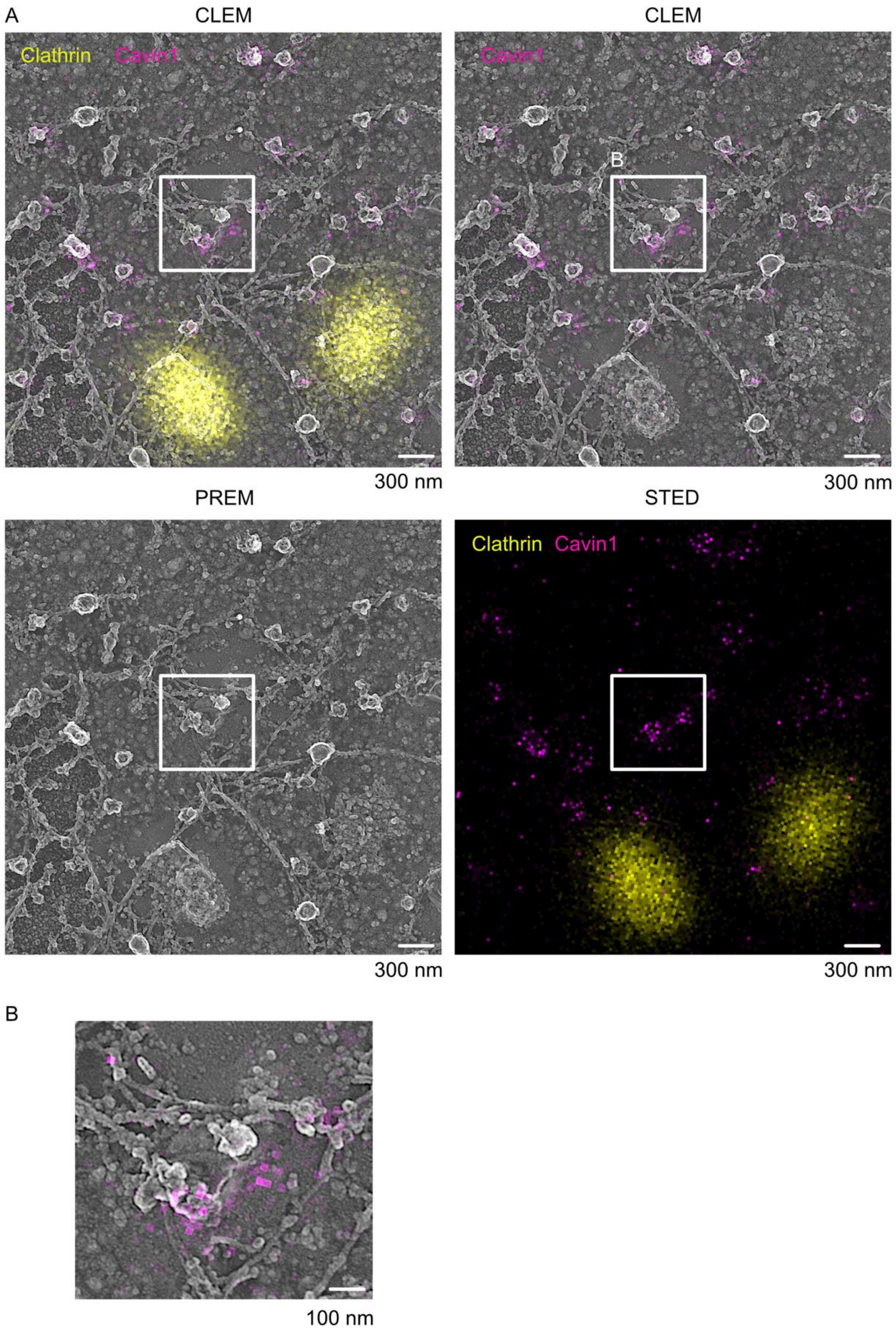

Supplementary Figure S6: Structural details in CLEM images.

(A) Representative overview CLEM image showing a MEF plasma membrane sheet. Clathrin (yellow) and cavin1 (magenta) were labelled.

(B) Detailed inspection of zoom box in A illustrates that middle STED fluorescence is not correlating with underlying membrane domain. The corresponding caveola is shifted (arrow).

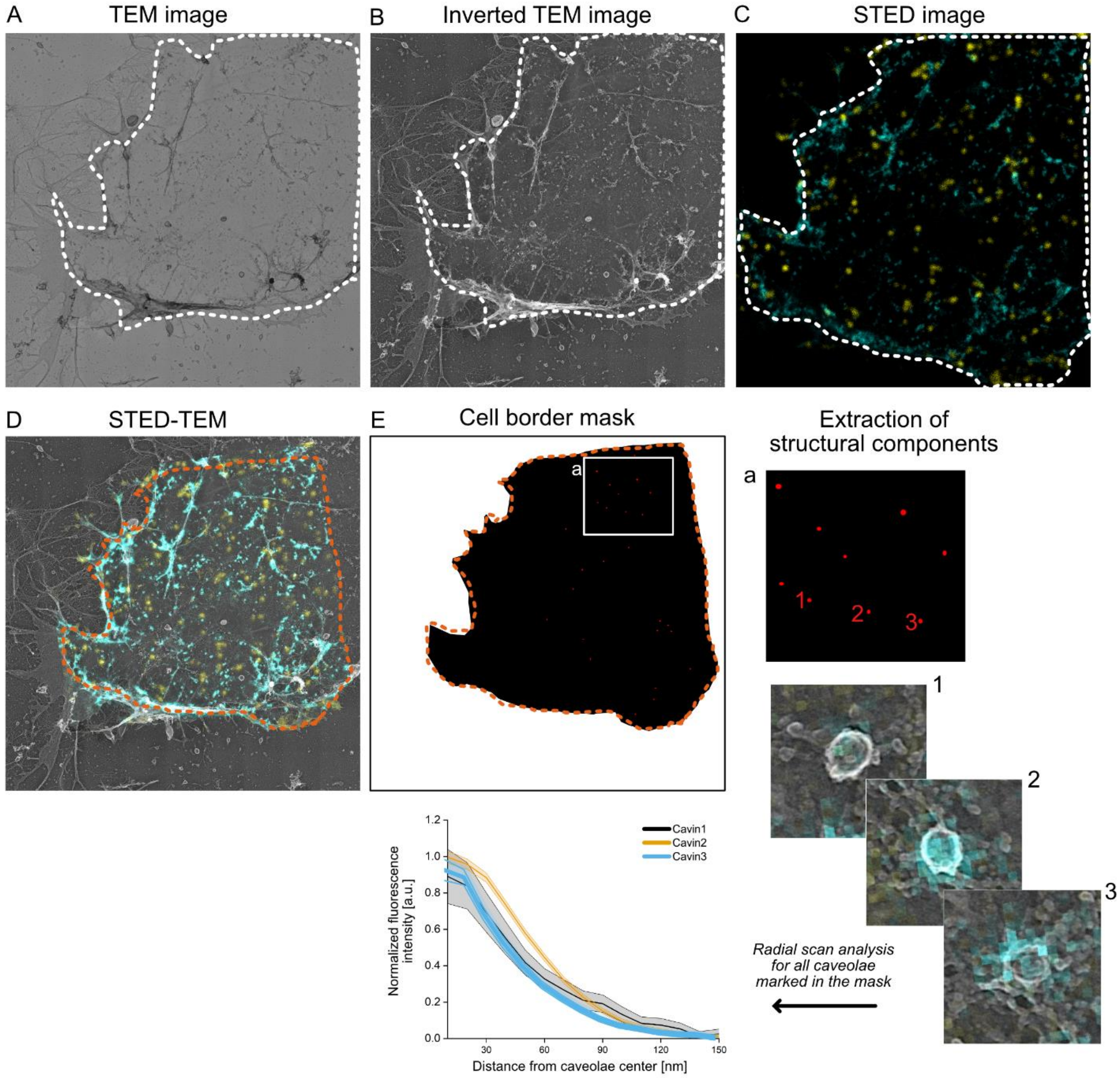

Supplementary Figure S7: Analyzing CLEM images.

(A-B) TEM images showing unroofed cell region (A). After inverting the TEM image, the cell edge (marked by white line) and structural details are detectable easily.

(C) Corresponding STED image shows fluorescence intensity for clathrin (yellow) and cavin2 (cyan). Cell edge is depicted in white.

(D) After correlation of inverted TEM and STED image the CLEM image is generated. Cell edge is marked in orange, clathrin in yellow and cavin2 in cyan.

(E) In ImageJ a cell mask is generated marking the unroofed cell area that was imaged by STED in black and outside the cell in white (cell edge in orange). Within the mask structural components (e.g. caveolae) can be marked, followed by extracting and stacking of the corresponding CLEM image parts and analyzing fluorescence intensities, size or occurrence of the investigated structures.
